# Recurrence of LV Thrombus in a Patient with Severe Ischaemic Cardiomyopathy Anticoagulated with Apixaban

**DOI:** 10.1155/2022/8156942

**Published:** 2022-07-21

**Authors:** Azhar Farooqui, Tin Lwin, Daniel Costa, Simon Hetherington, Raj Mattu

**Affiliations:** ^1^Department of Cardiology, Kettering General Hospital NS Foundation Trust, Rothwell Road, Kettering, NN16 8UZ, UK; ^2^University Hospitals of Leicester NHS Trust, Leicester, UK; ^3^Department of Cardiology, Castle Hill Hospital, UK; ^4^University of Leicester, Leicester, UK; ^5^University College London, London, UK

## Abstract

**Background:**

Anticoagulation with warfarin remains the mainstay treatment for left ventricular thrombi. Although successful thrombus resolution has been reported with direct oral anticoagulants' (DOACs') recurrence/progression while resuming Apixaban therapy is yet to be reported. *Case Presentation*. This case report describes left ventricular thrombus progression/recurrence in a patient anticoagulated with Apixaban undergoing implantable cardioverter defibrillator to cardiac resynchronisation therapy with defibrillator upgrade procedure. He was thereafter changed to warfarin. Contrast echocardiogram at 6 months follow-up did not identify the previously demonstrated mural thrombus.

**Conclusion:**

Prospective randomised control trials should be conducted in patients with left ventricular thrombus to compare anticoagulants, assess the efficacy and safety of DOACs, and evaluate uninterrupted DOAC versus transient withdrawal for cardiac device procedures.

## 1. Background

Left ventricular thrombus (LVT) complicates between 2.5 and 15% acute myocardial infarctions (MIs) [1]. Left ventricular (LV) failure also associates with LVT, especially amongst patients with left ventricular ejection fractions (LVEFs) less than 40% [2]. LVT relates to increased morbidity and mortality [2–4].

Anticoagulation with warfarin or low-molecular weight heparin (LMWH) remains the mainstay of treatment whilst successful thrombus resolution is reported with direct oral anticoagulants (DOACs) [2, 5]. Recurrence or progression of LVT after successful resolution while continuing Apixaban is yet to be reported. This case report describes LVT progression/recurrence in a patient anticoagulated with Apixaban undergoing ICD to CRT-D upgrade procedure.

## 2. Case Presentation

A 75 year-old gentleman was admitted for elective upgrade of his ICD to a CRT-D. He had a background of ischaemic cardiomyopathy, severe left ventricular systolic dysfunction (LVSD), ICD implant (2017), chronic obstructive pulmonary disease, hypothyroidism, glaucoma, and gout.

In 2017, he presented to the cardiology service with unexplained shortness of breath. Echocardiogram showed severe LVSD (LVEF < 30%) with extensive regional wall motion abnormalities (RWMAs) and akinesia of the apex. ECG showed sinus rhythm with narrow-complex QRS. His angiogram revealed significant coronary artery disease involving proximal chronic subtotal occlusion of the left anterior descending artery and focal tight stenosis of the midright coronary artery.

**Figure 1:**
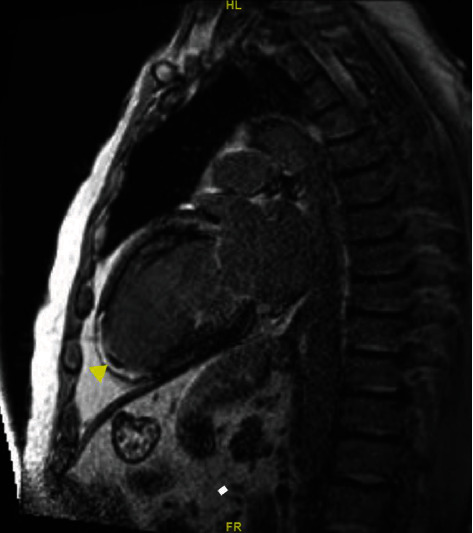
Delayed enhanced cardiac magnetic resonance image. Delayed enhanced cardiac MR (CMR) demonstrating an apical laminar thrombus (blocked arrow).

Cardiac magnetic resonance (CMR) demonstrated marked ischemic cardiomyopathy, severe LV dilation (EDD 62 mm, ESD 54 mm) and LVSD (EF 24%), transmural MI of midanteroseptum and true apex, partial MI of apical, inferior, anterior walls, and midinferoseptal and apical septal walls. The multidisciplinary team meeting (MDT) decided that surgical and percutaneous revascularisation were unsuitable. A small apical laminar thrombus was noted (Figure 1), whereupon the patient's dual antiplatelet therapy was changed to Apixaban 5 mg bd. A primary prevention ICD was implanted in 2017 for underlying ischaemic cardiomyopathy with severe LVSD. Optimisation of heart failure medications improved the symptoms from New York Heart Association class IV to II.

Repeat echocardiogram in 2019 showed unchanged severe LVSD; the previous apical laminar thrombus noted on CMR that was not identified on the echocardiogram. Given the akinetic apex, severe LVSD, and high risk for development of further intraventricular thrombus, Apixaban was continued.

**Figure 2:**
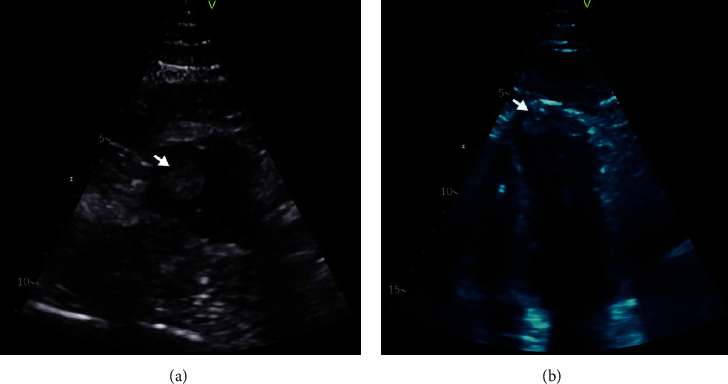
Post device upgrade ECHO. (a) Parasternal short axis view and (b) apical 4-chamber view of transthoracic echocardiogram demonstrating the left ventricular apical thrombus (arrow).

**Figure 3:**
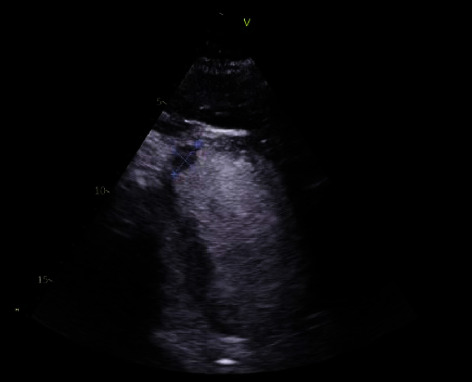
Contrast-enhanced transthoracic echocardiogram. Contrast ECHO demonstrating the left ventricular thrombus.

During review in November 2019, it was noted that his narrow-complex tachycardia had changed to right bundle branch block with QRS duration of >150 ms. He met the criteria for upgrade to a CRT-D and was admitted for device upgrade. Post procedure ECHO demonstrated a mural thrombus at the left ventricular apex (1.8 × 1.1 cm) (Figures 2 and 3). The patient throughout remained compliant with medications and in sinus rhythm; the only times Apixaban was withheld was 48 hours prior to ICD implantation (2017) and 48 hours prior to ICD upgrade to CRT-D during current admission. He was thereafter changed to warfarin. Contrast ECHO at 6 months follow-up did not identify the previously demonstrated mural thrombus.

## 3. Discussion and Conclusion

Anticoagulation with DOACs is increasingly utilised as off-label alternatives to warfarin in managing patients with LVT, with several case reports and observational studies demonstrating successful resolution on follow-up imaging [6].

In a retrospective observational study involving echocardiographic surveillance of 108 patients receiving long-term anticoagulation after diagnosis of LVT [2], complete thrombus resolution was found on follow-up ECHO at 1-year in all patients that received a DOAC, in 75% of patients on warfarin and 40% of those on LWMH. Interpretation must remain cautious as significantly fewer patients received DOAC therapy compared to warfarin and LMWH treatment groups (3.7% vs. 87% vs. 9.3%, respectively).

In a further study where 35 of 52 patients (67%) treated with DOAC for LVT proceeded to imaging follow-up, 83% had complete resolution of LVT with mean duration to complete resolution being 264 days [5]. The authors of this study recommended limited interpretation in duration to LVT resolution given nonstandardised timing of their follow-up echocardiograms.

A systematic review of thirty articles involving patients diagnosed with LVT and treated with DOACs found that thrombus resolution rates were 81%, 100%, and 88.9% for rivaroxaban, apixaban and dabigatran, respectively, with a median time to resolution of 40, 36, and 24 days, respectively [6].

Little is published on formation of new LVT whilst taking DOAC therapy, with one article describing this at the time of our report. Degheim et al. described a patient who developed LVT whilst on rivaroxaban for atrial fibrillation with subsequent subacute parietal lobe infarction [7].

CMR is superior to ECHO (with or without contrast) at detecting LVT [8]. It is possible the LV laminar thrombus found in our patient at CMR may not have completely resolved and went undetected on follow-up echocardiograms.

It raises the prospect that whether transiently withholding Apixaban for 48 hours prior to device upgrade may be a factor in formation or extension into the mural LV thrombus subsequently identified, given our patient's significantly impaired LV and akinetic apex.

Predisposition to significant thrombophilia appears unlikely in our patient by observations of no personal or family history of clinical thromboembolism and background coagulation profiles (×3) within normal limits.

Our observations raise the question as to whether patients diagnosed with LVT having underlying severe LVSD, significant RWMAs, or akinetic/aneurysmal apex should continue anticoagulation rather than withholding it prior to device upgrade procedures. In the event uninterrupted anticoagulation emerges as beneficial in LVT, it also remains to be determined as to what form this should take.

The BRUCE CONTROL 2 study [9] found no statistically significant difference in formation of clinically important hematoma postpacemaker or ICD-related procedures with continued compared to interrupted use of DOAC therapy.

The importance of the minimising risk of LVT formation is evidenced from patients with LVT having significantly increased risk of major adverse cardiovascular events (MACEs) and death, as highlighted in a present report from a single-centre French study examining 159 patients with LVT for effects of anticoagulation therapy on patient outcomes [4]. They received vitamin K antagonists (48.8%) followed by parenteral heparins (27.7%) and DOACs (22.6%). Antiplatelet therapy was also continued in 67.9% of patients. Total regression of LVT was observed in 62.3% and independently correlated with a smaller baseline thrombus area and a nonischemic cardiomyopathy.

With median follow-up of 632 days, 35.4% of patients with total LVT regression and 40.0% of patients with persistent LVT experienced MACE. Compared with other patients, those with LVT regression had lower risk of mortality, while anticoagulation therapy for more than 3 months and LVEF ≥35% were independently associated with lower rates of MACE.

At a median follow-up with ECHO of approximately 1 year, 76% of thrombi had regression from baseline. Independent predictors of complete resolution were nonischemic cardiomyopathy and smaller thrombus size at baseline.

While patients with LVT are at increased risk of adverse outcomes, there is also a lower mortality risk in patients showing LVT regression.

Nonpharmacologic mechanisms have also been suggested to aid the formation or progression of left ventricular thrombi in patient with aneurysmal apex. Fakhry and Siddique [10] suggested biventricular pacing-promoted ballooning of the left ventricular apex in their patient, leading to nonlaminar flow, stasis, and eventually leading to the formation of LVT.

In conclusion, prospective randomised control trials should be conducted in patients with LV thrombus to compare anticoagulants, assess the efficacy and safety of DOACs, and evaluate uninterrupted DOAC versus transient withdrawal for cardiac device procedures. This data may yield factors that enable individualised anticoagulation regime's periprocedure in LV thrombus.

### 3.1. Learning Objective

In this case report, the authors highlight to be mindful that LVT may propagate/recur in patients on DOAC therapy and transient withholding of DOAC therapy for cardiac device procedures may increase possible risk.

## Data Availability

All relevant data supporting the conclusions of this article are included within the article.

## Abbreviations

LVT: Left ventricular thrombus
LMWH: Low molecular weight heparin
DOAC: Direct oral anticoagulants.
ICD: Implantable cardioverter defibrillator
CRT-D: Cardiac resynchronisation therapy with defibrillator
LVSD: Left ventricular systolic dysfunction
RWMA: Regional wall motion abnormalities
ECHO: Echocardiogram
CMR: Cardiac magnetic resonance
MACEs: Major adverse cardiovascular events.
